# Focused ultrasound versus the loop electrosurgical excision procedure to treat women with cervical high-grade squamous intraepithelial lesions under 40: a retrospective study

**DOI:** 10.1186/s12885-024-11938-y

**Published:** 2024-02-03

**Authors:** Linlin Xiao, Xu Dong, Jiangchuan Sun, Xuerui Zhang, Qing Feng, Shufang Chang

**Affiliations:** Department of Obstetrics and Gynecology, The Second Affiliated Hospital of Chongqing Medical University, No. 74 Linjiang Road, Yuzhong District, Chongqing, 400000 China

**Keywords:** Focused ultrasound, Loop electrosurgical excision procedure, High-grade squamous intraepithelial lesions, HPV, Efficacy

## Abstract

**Background:**

This study aimed to compare the efficacy of focused ultrasound (FUS) and the loop electrosurgical excision procedure (LEEP) for the treatment of cervical high-grade squamous intraepithelial lesions (HSILs) among women of reproductive age.

**Methods:**

Case records of patients aged < 40 years who were treated for cervical HSILs using either FUS or LEEP from September 1, 2020 to May 31, 2022 were retrospectively reviewed. Patients were followed up for cure, recurrence, human papillomavirus (HPV) clearance, and complications within 1 year of treatment. Odds ratios and 95% confidence intervals were determined using univariate and multivariate logistic regression models to analyze the association between disease evidence or HPV clearance and treatment modalities or other covariates.

**Results:**

Of the 1,054 women who underwent FUS or LEEP, 225 met our selection criteria. Among the selected women, 101 and 124 received FUS and LEEP, respectively. There was no significant difference between the FUS and LEEP groups in the cure rate during the 3–6 months of follow-up (89.11% vs. 94.35%, *P* = 0.085) and recurrence rate during the 6–12 months follow-up (2.22% vs. 1.71%, *P* = 0.790). Both groups exhibited enhanced cumulative HPV clearance rates; however, the rates were not significantly different between the FUS and LEEP groups (74.23% vs. 82.79%, *P* = 0.122 during the 3–6 months follow-up; 84.95% vs. 89.17%, *P* = 0.359 during the 6–12 months follow-up). Furthermore, the incidence of complications caused by the FUS and LEEP techniques was comparable (5.0% vs. 5.6%, *P* = 0.818).

**Conclusions:**

We found that FUS and LEEP have similar efficacy, safety, and reliability in treating women (aged < 40 years) with HSILs.

## Background

Cervical cancer-related mortality is a public health concern among women, especially those living in low- and middle-income countries (LMICs) [1]. This cancer has a prolonged precancerous phase known as high-grade squamous intraepithelial lesions (HSILs). Persistent high-risk human papillomavirus (HR-HPV) infection is the primary cause of cervical precancer and cancer [2], which can be effectively prevented through high-quality screening and timely treatment of HSILs [3].

There is sufficient evidence for the development of HSILs and/or cervical cancer due to unhealthy sexual habits, the lack of immunization, immunosuppression, and persistent HR-HPV infections. Meanwhile, accumulating evidence suggests that factors such as duration of HPV persistence, positive margins of cervical conisation specimens, menopause, immunosuppression, and preoperative lesions involving the four quadrants are predisposing factors of HSIL recurrence [4–7]. Timely identification of these high-risk patients enables risk stratification, individualized management, and the development of follow-up strategies.

Treatment options for HSILs can be categorized as excision and ablation. Cervical conization, which includes cold knife conization and loop electrosurgical excision procedure (LEEP), is the preferred treatment method. As an outpatient procedure, LEEP is the first choice of treatment in high-resource settings because it has high efficacy and can provide a tissue specimen for pathologic examination [8]. Although excisional methods are widely used, they are associated with an increased risk of adverse reproductive outcomes, including preterm delivery and perinatal mortality [9, 10].

Owing to its low technical requirements, ablation is considered a simpler procedure than LEEP. Ablation includes cryotherapy, laser ablation, and thermal ablation. Furthermore, excisional techniques are not overwhelmingly superior to ablation techniques for the eradication of HSILs [11, 12]. Moreover, women who underwent ablation therapy were not at a high risk of preterm delivery (i.e., births that occurred after the procedure) [9, 10]. Owing to their concern regarding the high likelihood of cervical incompetence, women of reproductive age with plans for future conception prefer ablation as a treatment modality.

Focused ultrasound (FUS) is a new, eco-friendly, and noninvasive ablative technique. The high clinical efficacy of FUS for treating chronic cervicitis and cervical lesions has been previously demonstrated [13–17]. Qin et al. [13] demonstrated that FUS has a higher HPV elimination rate among women aged < 30 with HPV-related HSILs than LEEP; however, they did not evaluate the therapeutic efficacy and safety of FUS. Owing to the increasing marital and childbearing age and three-child policies in China, the number of women aged > 30 years with plans for future conception has increased. Therefore, the application of FUS in patients with HSIL who are of reproductive age warrants further investigation.

Through the evaluation and comparison of the efficacy and safety of the two treatments, this study aimed to provide evidence-based recommendations for the optimal management of HSILs in women of reproductive age.

## Methods

This was a retrospective cohort study of women (< 40 years old) with biopsy-confirmed HSILs who were treated with either FUS or LEEP at the Second Affiliated Hospital of Chongqing Medical University from September 1, 2020 to May 31, 2022. All patients consented to the review of their medical records according to the ethical approval guidelines (Ethics Committee of the hospital reference NO. 2022170).

Data were obtained from the medical records of patients who met the following criteria: patients aged 21–40 years with biopsy-proven HSILs (congruent with cytology results) who were treated with either FUS or LEEP; no prior history of HSILs; no evidence of underlying cancer or glandular epithelial lesions in cytology, biopsy, or colposcopic examination, especially in patients infected with HPV 16/18; satisfactory colposcopic examination results (complete visualization of the squamocolumnar junctions and entire lesions); lesions neither involving > 75% of the ectocervix nor extending to the endocervix or vagina; negative findings on endocervical curettage; and patients who completed both 3–6 months and 6–12 months of follow-up with complete data (HPV testing, thin-layer liquid-based cytology technology [TCT], and colposcopy in addition to cervical biopsy if necessary) until May 31, 2023. The exclusion criteria for patients in this study were as follows: (1) pregnancy, (2) previous ablative or excisional treatment for cervical HSIL, (3) current gynecological infection, and (4) significant medical illness that may lead to medical risk.

According to the guidelines for the management of abnormal cervical cancer screening tests [18], patients with abnormal screening test results were recommended to undergo colposcopy for further evaluation. The abnormal screening test results included persistent HR-HPV positive, HPV 16/18 positive, HPV-positive atypical squamous cell of undetermined significance (ASC-US), low-grade squamous intraepithelial lesion (LSIL), atypical squamous cells cannot exclude HSIL (ASC-H), HSIL, atypical glandular cells (AGC), adenocarcinoma in situ (AIS), squamous cell carcinoma (SCC), and adenocarcinoma. For suspected lesions, colposcopy-directed biopsy and/or cervical curettage was performed. Samples were recorded using the two-tier terminology recommended by the 2014 World Health Organization (WHO) classification of female genital tumours [19]. Results from TCT, colposcopy, and histological evaluation of the biopsies were used to make the final diagnosis, and determine appropriate management after consultation between practitioners and patients. An educational pamphlet about treatment procedures and potential adverse effects or complications was given to patients immediately before the procedure to make them aware of FUS or LEEP and obtain consent from the patients.

FUS ablation was performed using the Model-CZF300 Ultrasound Therapeutic Device for Gynecology (Chongqing Haifu Medical Technology Co., Ltd., Chongqing, China) under colposcopic guidance, as described in our previous study [15]. The treatment probe was covered with a coupling gel and applied over the transformation zone and lesions at a velocity of 2–5 mm/s for approximately 3–5 min until the surface of the cervix shrank.

LEEP was performed using the DGD-300B-2 electrosurgical apparatus (Beilin Electronics Co., Ltd., Beijing, China) under local anesthesia. Following the standard practice, resection was performed under colposcopic guidance using loop electrodes with an appropriate diameter according to the type of transformation zone and extent of lesions. Hemostasis was achieved by electrocoagulation using a ball electrode.

Postprocedural care instructions were provided to the patients. All patients were counseled to abstain from using tampons, vaginal douching, and sexual intercourse for at least 4 weeks. Furthermore, the precautions and situations for seeking medical advice, including heavy vaginal bleeding, foul-smelling or pus-like discharge, fever for > 2 days, or severe lower abdominal pain, were discussed.

All patients were advised to present for follow-up examinations after 3–6 months and 6–12 months of FUS or LEEP treatment. Co-testing with TCT, HPV typing test, and colposcopy were performed at each visit. If one of the test results was abnormal, any identified abnormal areas or endocervical sampling was recommended. A follow-up appointment was scheduled to review the pathology and plan for further evaluation as per the guidelines.

The primary post hoc outcome was the efficacy of the two treatment methods, which was determined based on the absence of disease evidence, HPV clearance, and recurrence. Irrespective of HPV clearance, the criteria for defining “no evidence of disease” during the follow-up period included negative screening results for cytology and colposcopy and histological confirmation of the absence of disease if a biopsy sample was obtained. HPV clearance during the follow-up period was defined as the absence of the same specific HPV type that was detected at the time of diagnosis. Recurrence was defined as a subsequent positive biopsy result for HSILs or higher during the 6–12 months follow-up among patients who were considered disease free at the first follow-up. The additional post hoc outcome was the safety of the two treatments, which was evaluated by assessing the number of complications caused by the procedures. Severe pain or cramps requiring medication during or after treatment, vaginal discharge or bleeding requiring a hospital visit, infection requiring antibiotics, and cervical stenosis requiring dilation were considered as complications.

All data were analyzed using SPSS version 26 (IBM, Armonk, NY, USA). The Mann–Whitney U-test was performed for continuous variables with a skewed distribution, whereas Fisher’s exact test was used for analyzing categorical variables. Odds ratios and 95% confidence intervals for no evidence of disease or HPV clearance associated with the treatment modalities and other covariates (age, educational background, contraception, lifetime sexual partners, parity, and HPV status) were estimated using univariate and multivariate logistic regression models. A *P* value of < 0.05 was considered statistically significant.

## Results

**Fig. 1:**
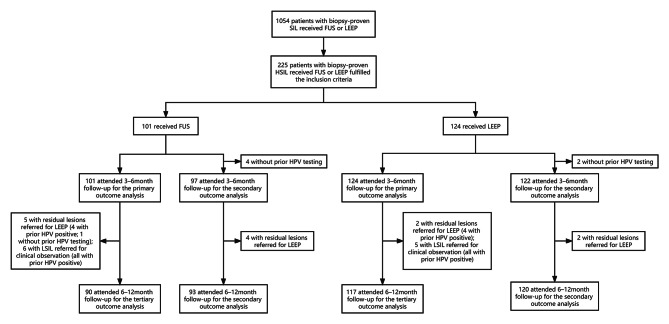
The study flowchart

Hospital records of 1,054 patients with cervical diseases who were treated with FUS or LEEP from September 1, 2020 to May 31, 2022 were reviewed. A total of 380 patients with biopsy-confirmed HSILs under the age of 40 years were identified. Among these patients, 101 and 279 received FUS and LEEP, respectively. However, in the LEEP group, there were 155 patients did not meet the inclusion criteria. Therefore, a total of 225 patients with histologically proven HSILs met our selection criteria, among whom 101 (44.9%) and 124 (55.1%) underwent FUS and LEEP, respectively. The enrollment profile of this study is shown in Fig. 1.

Table 1 presents the characteristics of the 225 patients. The distribution of age, used contraceptive method, total lifetime sexual partners, or parity were not balanced between the FUS and LEEP groups. However, other baseline characteristics were similar. The median age of the LEEP group was higher than that of the FUS group (*P* = 0.000). Compared with patients treated with LEEP, those treated with FUS preferred condoms for contraception (75.25% vs. 58.06%) and had more lifetime sexual partners (*P* = 0.000) but less parity (*P* = 0.000).

**Table 1:** Baseline characteristics by treatment group

		FUS(*n* = 101)	LEEP(*n* = 124)	U/***X***^**2**^	***P***
Age	Median (lower quartile, upper quartile)	26.91(24.00,31.00)	34.25(32.00,37.00)	*U* = 9.610	0.000
	range	18–38	21–40		
Educational background	<University	31(30.69%)	47(37.90%)	1.623	0.203
	≥University	70(69.31%)	77(62.10%)		
Contraception	Condom	76(75.25%)	72(58.06%)	11.999	0.002
	Others^*^	10(9.90%)	32(25.81%)		
	None	15(14.85%)	20(16.13%)		
Lifetime sexual partners	1–2	69(68.32%)	106(85.48%)	13.483	0.000
	≥ 3	32(31.68%)	18(14.52%)		
Parity	0	73(72.28%)	17(13.71%)	119.226	0.000
	1	26(25.74%)	74(59.68%)		
	≥ 2	2(1.98%)	33(26.62%)		
HPV status	HPV16/18 infection	50(49.51%)	53(42.74%)	3.435	0.179
	Non-HPV16/18 infection	47(46.53%)	69(55.65%)		
	Negative	0(0.00%)	0(0.00%)		
	Unknown	4(3.96%)	2(1.61%)		

*Others include intrauterine device, contraceptives, subdermal arm implant, tubal ligation and coitus interruptus

Table 2 shows that the difference between the cure rate during the 3–6 months follow-up (89.11% vs. 94.35%, *P* = 0.085) or the recurrence rate during the 6–12 months follow-up (2.22% vs. 1.71%, *P* = 0.790) of the two therapies was not significant. Furthermore, both groups showed enhanced cumulative HPV clearance rates without significant differences (74.23% vs. 82.79%, *P* = 0.122 during the 3–6 months follow-up; 84.95% vs. 89.17%, *P* = 0.359 during the 6–12 months follow-up; Table 2).

**Table 2:** Efficacy of FUS and LEEP

		FUS	LEEP	***X*** ^**2**^	***P***
3–6 months					
	Over all participants followed up	101	124		
	no evidence of disease	90(89.11%)	117(94.35%)	2.970	0.085
	HPV-positive at baseline participants followed up	97	122		
	clearance of HPV	72(74.23%)	101(82.79%)	2.386	0.122
6–12 months					
	No evidence of disease at 3-6month follow-up participants followed up	90	117		
	recurrent disease	2(2.22%)	2(1.71%)	0.071	0.790
	HPV-positive at baseline participants followed up	93	120		
	clearance of HPV	79(84.95%)	107(89.17%)	0.843	0.359

The effects of various factors on the cure rate during the 3–6 months follow-up and HPV clearance rate during the 6–12 months follow-up are shown in Tables 3 and 4, respectively. We found that patient’s age, educational background, contraception method, number of sexual partners, parity, and HPV status were not significantly associated with the cure rate or HPV clearance rate. After adjustments, FUS and LEEP still exhibited similar cure rates during the 3–6 months follow-up and HPV clearance rates during the 6–12 months follow-up.

**Table 3:** Logistic regression analysis for determining patient characteristics associated with cure rates during the 3–6-months follow-up

		Univariate analysis		Multivariate analysis	
Variable		OR(95%CI)	***P*** Value	OR(95%CI)	***P*** Value
Treatment	FUS	0.490(0.182–1.313)	0.156	0.407(0.097–1.708)	0.219
	LEEP	1		1	
Age		0.995(0.907–1.093)	0.921	0.867(0.749–1.003)	0.055
Educational background	<University	1.067(0.384–2.961)	0.901	1.040(0.345–3.137)	0.944
	≥University	1		1	
Contraception	Condom	0.282(0.036–2.216)	0.229	0.293(0.036–2.415)	0.254
	Others	0.382(0.038–3.850)	0.415	0.316(0.030–3.373)	0.340
	None	1		1	
Lifetime sexual partners	1–2	1.000(1.000−3.185)	1.000	0.746(0.210–2.654)	0.651
	≥ 3	1		1	
Parity	0	0.435(0.091–2.072)	0.296	0.301(0.043–2.125)	0.228
	1	1.152(0.213–6.222)	0.870	1.196(0.203–7.051)	0.843
	≥ 2	1		1	
HPV status	HPV16/18 infection	2.089(0.219–19.885)	0.522	1.548(0.145–16.473)	0.717
	Non-HPV16/18 infection	2.700(0.281–25.977)	0.390	2.312(0.210−25.434)	0.493
	Negative^*^	-	-	-	-
	Unknown	1		1	

*There was no HPV-negative patient

**Table 4:** Logistic regression analysis for determining patient characteristics associated with HPV clearance rates during the 6–12-months follow-up

		Univariate analysis		Multivariate analysis	
Variable		OR(95%CI)	***P*** Value	OR(95%CI)	***P*** Value
Treatment	FUS	0.644(0.290–1.428)	0.279	1.561(0.487–5.004)	0.454
	LEEP	1		1	
Age		0.944(0.874–1.021)	0.148	1.032(0.915–1.165)	0.607
Educational background	<University	1.672(0.676–4.135)	0.266	1.318(0.507–3.424)	0.571
	≥University	1		1	
Contraception	Condom	0.710(0.228–2.210)	0.554	0.840(0.253–2.791)	0.776
	Others	2.452(0.421–14.284)	0.319	2.129(0.346–13.086)	0.415
	None	1		1	
Lifetime sexual partners	1–2	1.175(0.467–2.952)	0.732	0.793(0.295–2.131)	0.646
	≥ 3	1		1	
Parity	0	0.129(0.016–1.009)	0.051	0.122(0.013–1.162)	0.067
	1	0.275(0.034–2.235)	0.227	0.253(0.030–2.134)	0.206
	≥ 2	1		1	
HPV status	HPV16/18 infection	2.472(0.249–24.561)	0.440	2.170(0.200−23.520)	0.524
	Non-HPV16/18 infection	1.347(0.142–12.760)	0.795	1.096(0.105–11.440)	0.939
	Negative^*^	-	-	-	-
	Unknown	1		1	

*There was no HPV-negative patient

Almost all patients in both groups reported no or minimal discomfort during or immediately after the procedure. Twelve patients developed complications related to the procedures: 5 of 101 (5.0%) patients in the FUS group and 7 of 124 (5.6%) patients in the LEEP group (*P* = 0.818). Among these patients, eight experienced heavy postoperative bleeding requiring vaginal gauze packing (FUS, *n* = 3; LEEP, *n* = 5), two suffered moderate postoperative lower abdominal cramps and required painkillers (FUS, *n* = 1; LEEP, *n* = 1), one patient receiving FUS had a localized cervical infection that required treatment with oral antibiotics, and one patient receiving LEEP had a cervical stenosis that required dilation 2 months after the procedure. None of these complications were life-threatening, and no patients required hospitalization.

## Discussion

Appropriate management of HSILs is crucial for the success of screening programs aimed at reducing the incidence of cervical cancer. Unfortunately, the screening of HSILs in LMICs is not quite effective partly due to the lack of appropriate treatment available for women with HSILs. The major challenge in screening HSILs is the lack of well-trained medical personnel who can perform colposcopy and provide treatment in primary healthcare facilities [1, 20]. The removal of HSILs via LEEP is an effective and safe treatment option, with low recurrence rates and high HPV clearance rates [12]. However, compared with ablation, LEEP requires highly skilled medical personnel and expensive equipment, produces odor and possible viral-laden smoke, and increases the risk of preterm labor in subsequent pregnancies in women of reproductive age.

FUS is a new, promising, noninvasive ablation-based therapeutic modality for cervical lesions. Similar to other ablative therapies, it does not provide tissue samples for further histological assessment. However, compared with other ablative therapies, FUS is an “inside–out” treatment method with a remarkable penetration depth of 3–6 mm [14], which exceeds the reported average depth of cervical intraepithelial neoplasia grade 3 (CIN3) (1.4 mm) [21]. From a practical perspective, FUS offers several advantages over conventional treatment methods. First, FUS equipment is more economical than other surgical tools, making it a cost-effective option for patients and healthcare providers. Second, the duration of operative training required to perform FUS procedures is shorter than that required to perform traditional treatment methods. Therefore, medical professionals can quickly learn to perform FUS and administer quality treatments to patients. Lastly, FUS offers conformal therapy, which is achieved by moving the probe. Previous studies have confirmed that the effectiveness, acceptability, and safety of FUS for the treatment of HPV-related cervicitis or cervical lesions [13–17]. Improvements in the availability and accessibility of precancer treatment can facilitate the use of FUS to treat patients with HSILs in low-resource settings, which can be appropriately performed by trained mid-level providers in primary healthcare services.

In this retrospective cohort study, patients in the LEEP group had higher median age and parity than those in the FUS group, which is consistent with observations made in clinical practice. Owing to the inherent biases of the retrospective study design, selection bias may have influenced the reported results. Therefore, we performed univariate and multivariate logistic regression analyses to reduce possible confounding factors. To ensure the comparability of the FUS and LEEP groups, we analyzed factors that may affect the treatment efficacy and HPV clearance using logistic regression. Both univariate and multivariate logistic regression analyses showed that no relevant factors significantly affected the cure and HPV clearance rates. However, future prospective randomized controlled trials are warranted to compare the efficacy of these two therapeutic approaches.

In terms of efficacy, the cure and recurrence rates of HSILs were comparable between the two treatment methods. The cure rate of LEEP in our study was slightly higher than that of FUS, although the difference was not statistically significant. The disease-free incidence was also higher in our study than that reported in most previous studies [22–25];  however, the recurrence rate of LEEP was similar [11, 22]. Differences in the study methods, follow-up periods, and selection criteria may account for this discrepancy. Regarding FUS efficacy, our results showed that FUS was at par with LEEP, with a cure rate of 89.11% during the 3–6 months follow-up and a recurrence rate of 2.22% during the 6–12 months follow-up. These results are consistent with the effectiveness of FUS determined in similar settings in previous studies, such as our previous studies that reported cure rates of 88.90–89.70% [14–15] and study conducted by Zhou et al. [17] that reported the cure rate of 82.80%. However, no recurrence was observed in patients with biopsy-proven HSILs receiving FUS in our previous studies. The higher recurrence rates in this study can be attributed to the difference in study methods and sample sizes. The role of persistent HR-HPV infection in determining the occurrence or recurrence of HSIL and cervical cancer is well established [2, 4–7]. Recently, a retrospective study for the first time reported that the risk of HSIL recurrence increased with the duration of HPV persistence (< 1 year). However, the persistence of HPV for > 1 year was not considered a risk factor [26].

Persistent HPV infection is the main risk factor for the persistence or recurrence of HSIL after treatment [27]. It has been shown that HPV test is highly sensitive, with a negative predictive value of approximately 98% for detecting recurrence at 6 months after treatment [28]. This indicates that women with negative HPV results after the treatment are at a low risk of recurrence [18]. Furthermore, persistent HPV infection might be an important cause of preterm labor [29, 30]. These findings indicate that patients with a negative HPV post-treatment status have a low risk of preterm labor. Therefore, the HPV post-treatment status should be considered when further determining the impact of FUS and LEEP on fertility outcomes. Notably, our study found no significant difference in HPV negativity rates after either FUS or LEEP (74.23% vs. 82.79% during the 3–6 months follow-up and 84.95% and 89.17% during the 6–12 months follow-up). The HPV of FUS was consistent with that reported in our previous studies [14–15], but it was lower than that reported by Qin et al. [13]. The lower clearance rate can be attributed to the difference in factors included in the inclusion criteria, such as age, type of transformation zone, and HPV status, as well as the difference in the criteria for clearance. These factors must be considered in future prospective, multicenter large-sample studies. Compared with the HPV clearance rate of 60–87% within 12 months of treatment reported in previous studies [22, 31–34], the accumulated clearance rate observed in our study was higher, possibly because of the differences in regions and age ranges of patients with HSILs analyzed.

This study found that both FUS and LEEP were safe and well-tolerated procedures for treating women with HSILs. Adverse effects and complications were rare and minor, and no significant difference was found in their incidence rates between the two treatments. Adverse effects or complications experienced by patients were majorly easily manageable, such as postoperative bleeding or lower abdominal cramps. This observation is consistent with those of previous reports on the safety of the two procedures [14–15, 17, 35]. Advancements in surgical techniques have led to the development of noninvasive or minimally invasive procedures that are viable alternatives for HSILs or cervical cancer [36, 37]. However, to better ensure the safety of treatment, clinical practitioners have been seeking other feasible and effective noninvasive or minimally invasive therapeutic approaches to further reduce the side effects or complications.

The main strength of this study is that it evaluated and compared the efficacy and safety of FUS and LEEP in treating HSILs in patients of reproductive age. In addition, this retrospective study analyzed the results obtained in actual clinical scenarios. Notably, it was found that younger women who desire to preserve their fertility preferred FUS over HSIL when the ablation criteria were met. We found that the cure, recurrence, and HPV clearance rates of the two HSIL treatment methods were similar. We also confirmed that both FUS and LEEP are safe and well-tolerated procedures for treating women with HSILs. However, the present study is limited by its retrospective design and the missing data on some patient characteristics. These limitations may affect the power of the study. However, these issues are common in clinical practice. Another major limitation was that women with HSILs who underwent FUS or LEEP were followed up for only 1 year after treatment, which was insufficient for evaluating their prognosis. Therefore, longer follow-ups should be implemented in future investigations to collect sufficient information regarding the prognosis of patients and tailor the most appropriate surveillance strategies for patients. Additionally, in our study, we found that cervical specimens were mainly recorded based on a two-tier terminology as recommended by the 2014 WHO classification of female genital tumours, which means the majority of HSILs were not specifically classified as CIN2 and CIN3. However, since the publication of 2020 WHO classification of female genital tumours [38], cervical specimens have been accurately recorded based on a two-tier terminology with specific grades (CIN1, CIN2 and CIN3). As the retrieval time for patient enrollment coincidentally covered the alternating period of the two editions of the WHO classification, we could not accurately classify HSILs as CIN2 or CIN3, which considerably differ in their ability for spontaneous regression. For a better assessment of the outcomes, the classification and distribution of CIN2 and CIN3 between the two treatment groups should be considered in further studies. Furthermore, the study was conducted in only one hospital with a small sample size. Furthermore, our population only included patients aged < 40 years; thus, our results cannot be generalized for all age clusters. Owing to the small population size, the results could not be generalized to the entire population. Sampling should be expanded to include pregnant women to determine the impact of FUS and LEEP on fertility outcomes. Therefore, further prospective studies with a larger sample, multicenter design, and homogeneity of patients are required to confirm these results.

## Conclusions

Our study indicates the similarity of effectiveness, safety, and reliability between FUS and LEEP in treating women with HSILs aged < 40 years.

## Data Availability

The data that support the findings of this study are not publicly available due to privacy or ethical restrictions, but are available from the corresponding author upon reasonable request.

## Abbreviations

AGC: Atypical Glandular Cells
AIS: Adenocarcinoma In Situ
ASC-US: Atypical Squamous Cell of Undetermined Significance
ASC-H: Atypical Squamous Cells cannot exclude HSIL
CIN: Cervical Intraepithelial Neoplasia
FUS: Focused Ultrasound
HSIL: High-grade Squamous Intraepithelial Lesions
HPV: Human Papillomavirus
HR-HPV: High-risk Human Papillomavirus
LEEP: Loop Electrosurgical Excision Procedure
LMICs: Low- and Middle-income Countries
LSIL: Low-grade Squamous Intraepithelial Lesion
SCC: Squamous Cell Carcinoma
TCT: Thin-layer Liquid-based Cytology Technology
WHO: World Health Organization
